# Metabolic activity determines survival depending on the level of lymph node involvement in cervical cancer

**DOI:** 10.1186/s12885-022-09785-w

**Published:** 2022-07-23

**Authors:** Alejandra Martinez, Elodie Chantalat, Martina Aida Angeles, Gwénaël Ferron, Anne Ducassou, Manon Daix, Justine Attal, Sarah Bétrian, Amélie Lusque, Erwan Gabiache

**Affiliations:** 1 Surgical Oncology Department. Institut Claudius Regaud, Institut Universitaire du Cancer de Toulouse-Oncopole, Toulouse, France; 2 Cancer Research Center of Toulouse (CRCT), UMR 1037, INSERM, Toulouse, France; 3 Gynecology Department. Centre Hospitalier, Universitaire de Toulouse. Institut Universitaire du Cancer de Toulouse-Oncopole, Toulouse, France; 4 Radiotherapy Department. Institut Claudius Regaud, Institut Universitaire du Cancer de Toulouse-Oncopole, Toulouse, France; 5 Medical Oncology Department. Institut Claudius Regaud. Institut Universitaire du Cancer de Toulouse Oncopole, Toulouse, France; 6 Biostatistics Department. Institut Claudius Regaud, Institut Universitaire du Cancer de Toulouse-Oncopole, Toulouse, France; 7 Nuclear Medicine Department. Institut Claudius Regaud. Institut Universitaire du Cancer de Toulouse Oncopole, Toulouse, France

**Keywords:** Uterine Cervical Neoplasms, Lymphatic metastasis, Positron Emission Tomography Computed Tomography, Locally advanced cervical cancer, Fluorodeoxyglucose F18, Survival Analysis

## Abstract

**Background:**

To assess the impact of PET/CT functional parameters on survival, locoregional, and distant failure according to the most distant level of lymph node [^18^F]FDG uptake in patients with locally advanced cervical cancer (LACC).

**Methods:**

Retrospective study including 148 patients with LACC treated with concurrent chemoradiotherapy after PET/CT and para-aortic lymph node (PALN) surgical staging. Two senior nuclear medicine physicians reviewed all PET/CT exams and retrieved tumor and lymph node metabolic parameters: SUVmax, MTV, TLG. Oncological outcomes according to metabolic parameters and level of lymph node spread on PET/CT were assessed.

**Results:**

In patients without lymph node uptake on PET/CT, high MTV values of the cervical tumor were associated with DFS (HR = 5.14 95%CI = [2.15–12.31]), OS (HR = 6.10 95%CI = [1.89–19.70]), and time to distant (HR = 4.73 95%CI = [1.55–14.44]) and locoregional recurrence (HR = 5.18 95%CI = [1.72–15.60]). In patients with pelvic lymph node (PLN) uptake but without PALN uptake on [^18^F]FDG-PET/CT, high MTV values of the cervical tumor were associated with DFS (HR = 3.17 95%CI = [1.02–9.83]) and OS (HR = 3.46 95%CI = [0.96–12.50]), and the number of PLN fixations was associated with DFS (HR = 1.30 95%CI = [1.10–1.53]), OS (HR = 1.35 95%CI = [1.11–1.64]), and time to distant (HR = 1.35 95%CI = [1.08–1.67]) and locoregional recurrence (HR = 1.31 95%CI = [1.08–1.59]). There was no significant association between cervical tumor metabolic or lymph node metrics and survival outcome in patients with PALN uptake.

**Conclusions:**

Cervical MTV is more accurate than SUVmax to predict survival outcome in patients with locoregional disease confined to the pelvis and should be implemented in routine clinical practice. Prognostic value of metabolic metrics disappears with PALN uptake, which is associated with distant failure in nearly half of patients.

**Graphical Abstract:**

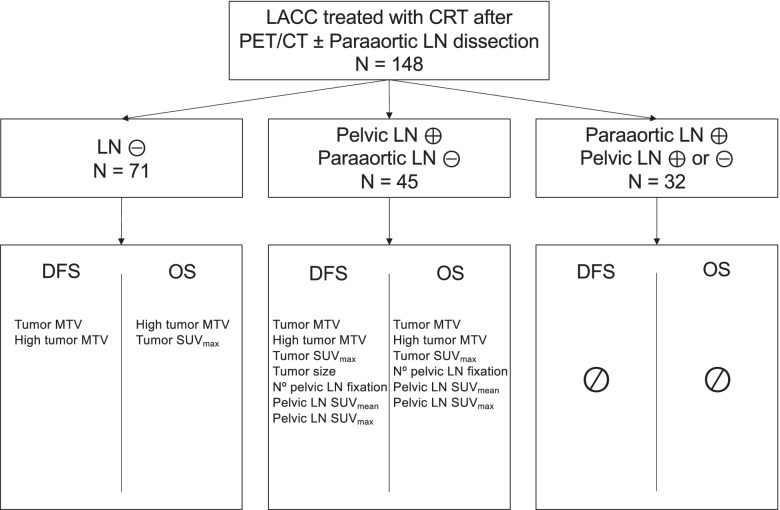

## Key points

- Metabolic tumor volume of the cervical tumor is more accurate than SUVmax to predict survival outcome in patients with locoregional disease confined to the pelvis without paraaortic lymph node uptake.
- Prognostic value of metabolic metrics disappears in patients with paraaortic lymph node uptake, which is associated with distant failure in nearly half of them.

## Background

Cervical cancer is one of the most common malignant diseases worldwide and is one of the most common causes of death among women [1]. Although cervical cancer is often curable if detected early, more than one third of patients present a locally advanced cervical cancer (LACC) at diagnosis in developed countries [2]. Among several prognostic factors, lymph node status is the most important, and patients with extension up to the paraaortic area have a 3-year survival rate of approximately 30%. Most recurrences in these patients are distant failures [3, 4]. Assessment of lymph node involvement with different imaging modalities was evaluated in a meta-analysis including 41 studies [5]. Results showed a higher overall diagnostic performance of positron emission tomography/computed tomography (PET/CT) in a per-patient and a region or node-based analysis. Sensitivity was 82%, 50%, and 56%, and specificity was 95%, 90%, and 91% for PET/CT, computed tomography (CT), and magnetic resonance imaging (MRI), respectively [5].

In previous studies, our group and others demonstrated that tumor and lymph node metabolic parameters are able to predict treatment response and recurrence risk in patients treated with surgery or chemoradiotherapy (CRT) for cervical cancer [6–9]. Even if tumor metrics correlate with the presence of lymph node involvement [10–13], the prognostic value of tumor metabolic activity is probably dependent on locoregional extension, and on the level of lymph node metastases. There are some series addressing this question, but they are relatively old and include patients with early- and advanced-stage cervical cancer, and with variable degrees of lymph node extension. The magnitude to which PET/CT tumor and lymph node functional parameters influence patients’ outcome in relation to lymph node extension is unclear.

We studied the influence of metabolic parameters according to the level of lymph node spread on LACC, and on locoregional and metastatic progression.

## Materials and methods

This retrospective study included patients with LACC (clinical FIGO stage 2009 IB2-IVA, except IIA1 without lymph node involvement) who received pre-therapeutic fluorodeoxyglucose [^18^F]FDG-PET/CT imaging at the French Referral Cancer Center from January 2006 through March 2015. The project was approved by the Institutional Review Board.

Preoperative work-up in all cases included physical examination, cervical biopsy, pelvic MRI, [^18^F]FDG-PET/CT, and laparoscopic paraaortic lymph node (PALN) retroperitoneal staging. Surgery began with a transperitoneal diagnostic laparoscopy to rule out occult carcinomatosis. A 10-mm port was inserted by open laparoscopy, and a 5-mm operative right lateral trocar was used to improve peritoneal evaluation. When peritoneal carcinomatosis was identified, the patient was excluded from the study and referred to palliative chemotherapy. If no anomaly was found, an extraperitoneal PALN dissection was performed through an extraperitoneal approach, as previously described [14]. During surgical staging, a frozen section was performed in cases of macroscopically suspicious lymph nodes. The surgical procedure was aborted if lymph node involvement was confirmed. Pelvic lymph nodes (PLN) were those situated in the pelvic region caudally to the common iliac bifurcation. PET/CT para-aortic lymph nodes included nodes from the bifurcation of the common iliac artery caudally to the left renal vein cranially. Para-aortic surgical dissection was performed using the same PET/CT anatomic limits, and included lympho-fatty tissue form the common iliac vessels, the aorta, aorto-caval space, and the vena cava.

Patients underwent pelvic with or without paraaortic external beam radiotherapy combined with chemotherapy. Radiotherapy was administered to the whole pelvic region in 25 fractions of 1.8 Gray (Gy) for a total dose of 45 Gy within 5 weeks. The paraaortic area also received 45 Gy in 25 fractions when PALN retroperitoneal staging was found to be positive. Concomitant chemotherapy with cisplatin 40 mg/m^2^ was administered weekly during radiotherapy for five courses. The treatment was then completed with additional pulse dose rate intracavitary brachytherapy for an equivalent total dose of 80–90 Gy. Before 2008, additional boosts up to an equivalent total dose of 65 Gy were sometimes given at the end of brachytherapy in the event of macroscopic lymph node and/or parametrial involvement. When Intensity-Modulated Radiation Therapy (IMRT) became available, a simultaneous integrated boost was performed on positive PLN at doses of 57.5 Gy in 25 fractions. PLN were considered positive when confirmed by pathology exam, or when there was a moderately to markedly deviation of the [^18^F]FDG uptake from the physiological distribution on pre-treatment PET/CT.

Follow-up included clinical examination of patients every four months for two years, and every six months for the following three years. Additional imaging was performed if clinically indicated using MRI for local evaluation and PET/CT for distant disease.

Exclusion criteria consisted of non-available images of [^18^F]FDG-PET/CT for double reading, distant metastasis at diagnosis, or peritoneal carcinomatosis found at laparoscopic examination of the abdominal cavity.

Medical data were extracted from computerized medical records and included demographics, clinical characteristics, imaging, surgical staging, histological findings, treatment and follow-up data, as well as recurrence and survival status at the end of the study.

### *[*^*18*^*F]FDG-PET/CT *modalities and review

Prior to any treatment, [^18^F]FDG-PET/CT was performed in the initial work-up according to the standardized institutional protocol. [^18^F]FDG-PET/CT whole-body images were obtained using a full-ring PET/CT scanner. Patients fasted for at least six hours before scanning. Blood glucose levels were checked before [^18^F]FDG injection, and injected dose and time between injection and acquisition were noted. If necessary, regarding bladder repletion and urinary activity, complementary pelvic acquisitions could be done after administering 20 mg of furosemide. PET data were reconstructed using an iterative, fully 3D algorithm with CT images for attenuation correction. A senior nuclear medicine physician expert in gynecologic cancer analyzed all [^18^F]FDG-PET/CT images in standard clinical fashion. All patients had a double-blinded review of metabolic parameters performed by another senior nuclear medicine specialist. Segmentation of cervical tumor volumes and PLN was done using General Electric AW server 3.0 software with an automatic thresholding at 40% of maximum standardized uptake value (SUVmax), following European Association of Nuclear Medicine (EANM) guidelines [15]. Manual correction was used in a few cases to exclude urinary tract activity, mostly in patients who had not received furosemide and whose bladder activity was equal or superior to tumor uptake. This was also the case when the AW Server automatic thresholding process was not suitable. For this modification, we used CT scan and visual uptake differences between tumor and urinary activity. Other tumor contours were not modified. Lymph nodes were considered as involved if they showed any uptake superior to background activity. The metabolic parameters studied for primary cervical tumor and/or for PLN when positive were as follows: SUVmax, mean standardized uptake value (SUVmean), metabolic tumor volume (MTV), total lesion glycolysis (TLG), and number of PLN fixations. The size of PLN was measured on CT imaging. The MTV and TLG of PLN used in our study were measured from the most [^18^F]FDG avid lesion, allowing for quick assessment by means of a procedure that can be used in daily clinical practice. A high MTV value was defined as a value above the median MTV of the whole cohort.

### Statistical analysis

Qualitative variables were described by frequencies and percentages and compared using the Chi-squared or Fisher’s exact test. Continuous variables were summarized by median and range (min–max) and compared using the Kruskal–Wallis test. All survival times were calculated from the initiation of CRT and were estimated by the Kaplan–Meier method with 95% confidence intervals (CI), using the following definitions of first event: loco-regional relapse for time to loco-regional relapse, metastatic relapse for time to distant metastasis, relapse or death for disease-free survival (DFS) and death for overall survival (OS). Patients who did not experience the event of interest were censored at their last follow-up. Univariate analyses were performed using the Cox proportional hazard model for continuous variables and the Log-rank test for qualitative variables. Hazard Ratios (HR) with their 95% CI based on the Cox proportional hazards model were calculated for each variable. All statistical tests were two-sided and a *p*-value < 0.05 was considered statistically significant. Statistical analyses were performed using the STATA software version 16 (StataCorp LLC, College Station, TX).

## Results

During the study period, 148 patients met the inclusion criteria. Clinical characteristics are presented in Table 1. Among the 148 patients, [^18^F]FDG-PET/CT showed no lymph node involvement in 71, 45 had exclusive PLN involvement on [^18^F]FDG-PET/CT, and there was PALN involvement with or without PLN involvement in 32. PALN dissection was performed in 133 patients and 15 were considered metastatic on the PALN based on pretreatment PET/CT results. The median number of PALN removed was 18 (range 5–48). One patient had macroscopic lymph node involvement confirmed by frozen section, so PALN dissection was aborted. The proportion of patients without PALN uptake on [^18^F]FDG-PET/CT and with pathology-proven lymph node involvement after surgical staging (false-negative rate) was 4.2% (3/71) in patients without PLN [^18^F]FDG-PET/CT uptake, and 24.4% (11/45) in patients with PLN [^18^F]FDG-PET/CT uptake. In total, 46 patients had PALN involvement: 3 in the negative FDG-PET/CT group, 11 in the positive PLN but negative PALN [^18^F]FDG-PET/CT group, and 32 in the positive PALN [^18^F]FDG-PET/CT group (15 only on imaging and 17 confirmed on PALN dissection). Among the 3 patients with negative PLN and positive PALN, one presented with micro-metastasis and the other two had macro-metastases. Among the remaining 28 patients with positive PALN confirmed histologically (11 patients with positive PLN but negative PALN [^18^F]FDG-PET/CT and 17 with positive PALN [^18^F]FDG-PET/CT), all had macro-metastases. Clinical characteristics, histological subtype, and clinical FIGO stage were similar between patients with and without PALN involvement.

**Table 1:** Patients’ characteristics (*n* = 148)

Clinical characteristics	n (%)
**Age (years)**, median (range)	50 (27–77)
**BMI (kg/m**^**2**^**)**, median (range)	23.2 (15.4–39.8)
**Histologic subtype**, n (%)	
Squamous	131 (88.5)
Adenocarcinoma	14 (9.5)
Adenosquamous	3 (2.0)
**Clinical FIGO stage (2009),** n (%)	
IB2-IIA^a^	36 (24.3)
IIB-IVA	112 (75.7)

*BMI* body mass index, *FIGO* International Federation of Gynecology and Obstetrics

^a^Patients with stage IIA were stage IIA2, or had pelvic lymph node involvement on preoperative imaging

### Metabolic parameters of cervical tumor and pelvic lymph nodes

Median values of cervical tumor SUVmax, MTV and TLG for the overall cohort were 14.8 (range 2.1–46.3), 39.2 cc (range 0.8–299.0) and 263.0 g/mL*cm3 (range 1.2–3181.0), respectively. Median values of cervical tumor SUVmax, MTV and TLG in patients without lymph node involvement on [^18^F]FDG-PET/CT were significantly lower than in patients with positive lymph nodes on [^18^F]FDG-PET/CT. Seventy-two patients had abnormal PLN uptake: 27 (37.5%) with positive PALN and 45 (62.5%) with negative PALN. Cervical tumor and PLN metabolic parameters according to lymph node status on PET/CT are displayed in Table 2.

**Table 2:** Tumor and pelvic lymph node metabolic activity according to lymph node status on PET/CT

	**PLN-/PALN-** median (range)	**PLN + /PALN-** median (range)	**PALN + ** median (range)	*p-*value
**Tumor metabolic activity**	***n* = 71**	***n* = 45**	***n* = 32**	
SUVmax	13.9 (2.1–46.3)	15.7 (7.3–34.4)	14.8 (6.0–23.9)	**0.008**
MTV (cc)	25.9 (0.8–162.0)	48.6 (8.8–127.0)	52.3 (10.5–299.0)	**0.001**
TLG (g/mL*cm^3^)	197.6 (1.2–2495.0)	432.7 (43.4–2204.0)	353.4 (42.2–3181.0)	**0.001**
**PLN metabolic activity**		***n* = 45**	***n* = 27**	
SUVmax	N/A	6.7 (1.7–23.9)	6.6 (1.8–16.1)	0.794
SUVmean	N/A	4.0 (1.3–12.6)	4.1 (1.6–9.0)	0.907
Ratio of PLN SUVmax/tumor SUVmax	N/A	0.40 (0.10–1.25)	0.49 (0.12–0.95)	0.467
MTV_PLN_ (cc)	N/A	1.9 (0.3–22.8)	2.4 (0.2–8.8)	0.646
TLG (g/mLxcm^3^)	N/A	6.8 (0.6–152.2)	10.4 (0.4–76.8)	0.831

*PLN* pelvic lymph node, *PALN* para-aortic lymph node, *N/A* not applicable

**Fig. 1:**
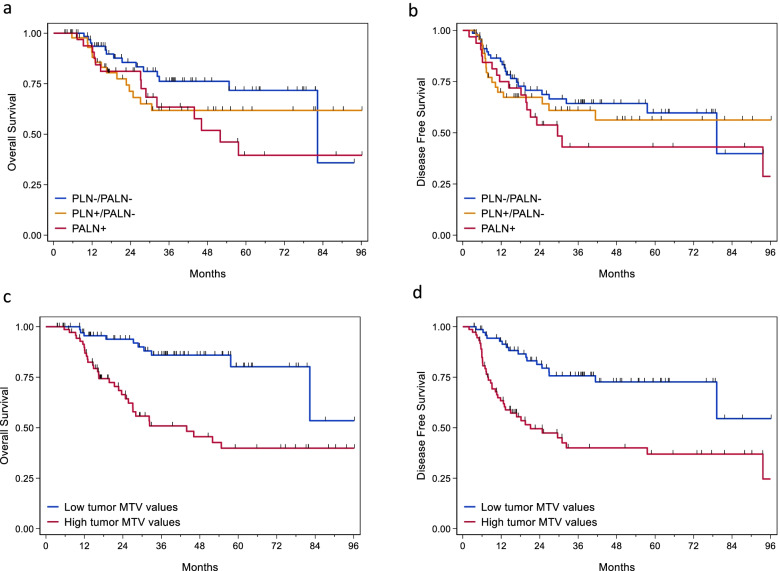
**a** Overall survival (OS) according to lymph node spread; **b **Disease-free survival (DFS) according to lymph node spread; **c **OS according to MTV values; **d **DFS according to MTV values

After a median follow-up of 39.3 months (95% CI 33.4–47.9), 42 patients (28.4%) died from the disease. During the study period, 55 patients (37.2%) recurred: 18 (32.7%) had a locoregional recurrence, 20 (36.4%) had distant metastases and 17 (30.9%) patients presented with both locoregional and distant disease. The 2- and 5-year OS rates were 80.2% (95% CI 72.0–86.2) and 59.8% (95% CI 48.4–69.5) respectively. Figure 1 shows Kaplan–Meier OS and DFS estimated curves according to lymph node spread on [^18^F]FDG-PET/CT and MTV values of cervical tumor.

In univariate analysis, FIGO stage IIB-IVA, pretherapeutic tumor size measured by MRI, tumor SUVmax, MTV of cervical tumor, PLN SUVmax, and the number of PLN with uptake at [^18^F]FDG-PET/CT were significantly associated with decreased DFS and OS (Table 3). In multivariate analysis, the only metabolic parameter that remained significantly associated with DFS in the whole cohort was the number of [^18^F]FDG-PET/CT PLN abnormal uptake. MTV of the cervical tumor and the number of PLN fixations were the only two metabolic parameters which remained significantly associated with OS.

**Table 3:** Univariate analysis for overall and disease-free survival

	**Overall Survival**		**Disease-Free Survival**	
	**HR (95%CI)**	***p*-value**	**HR (95%CI)**	***p*-value**
Age ≥ 55 vs < 55 years (ref.)	0.58 (0.28–1.17)	0.124	0.62 (0.35–1.12)	0.109
Histological subtype other than squamous vs squamous (ref.)	0.99 (0.35–2.78)	0.988	0.82 (0.33–2.05)	0.668
FIGO stage IIB-IVA vs IB2-IIA2 (ref.)	3.54 (1.26–9.94)	**0.010**	3.38 (1.45–7.88)	**0.003**
Tumor size on imaging (cm)	1.35 (1.11–1.64)	**0.003**	1.27 (1.08–1.50)	**0.004**
Tumor SUVmax	1.07 (1.03–1.12)	**0.001**	1.05 (1.01–1.09)	**0.012**
Tumor MTV (/10 cc)	1.10 (1.04–1.16)	** < 0.001**	1.07 (1.03–1.13)	**0.002**
PLN SUVmax	1.15 (1.05–1.26)	**0.002**	1.12 (1.03–1.21)	**0.008**
Number of PLN with uptake at FDG-PET/CT	1.19 (1.07–1.33)	**0.002**	1.17 (1.05–1.30)	**0.004**

*HR* Hazard Ratio, *CI* Confidence Interval, *FIGO* International Federation of Gynecology and Obstetrics, *SUVmax* maximum standardized uptake value, *MTV* metabolic tumor volume, *PLN* pelvic lymph node, *FDG-PET/CT* fluorodeoxyglucose-positron emission tomography/computed tomography

In the group of patients with no lymph node involvement on [^18^F]FDG-PET/CT, 15 patients (21.1%, 15/71) and 14 patients (19.7%, 14/71) had loco-regional and distant failures, respectively, compared to 9 loco-regional (28.1%, 9/32) and 14 distant failures (43.8%, 14/32) in patients with PALN involvement.

To evaluate the influence of metabolic parameters on survival and on locoregional and distant recurrence, we grouped patients by [^18^F]FDG uptake on the most distant level of lymph node activity. MTV and high MTV values of the cervical tumor were significantly associated with the risk of recurrence in patients without lymph node involvement on [^18^F]FDG-PET/CT. In patients with PLN uptake and without PALN uptake, tumor SUVmax, MTV, high MTV values, tumor size, number of PLN with uptake, PLN SUV mean and SUVmax were significantly associated with the risk of recurrence. No significant association was found between tumor or lymph node metabolic parameters and DFS in patients with PALN uptake with or without PLN uptake. The risk of death was significantly associated with tumor SUVmax and high MTV values in patients without lymph node involvement on [^18^F]FDG-PET/CT. Tumor SUVmax, MTV, high MTV values, number of PLN with uptake, PLN SUVmean and SUVmax were significantly associated with the risk of recurrence in patients with PLN uptake but without PALN uptake. No significant association between tumor metabolic or lymph node metrics and OS was found in patients with PALN uptake with or without PLN uptake (Table 4).

**Table 4:** Univariate analysis of overall and disease-free survival according to most distant level of lymph node uptake on [^18^F]FDG-PET/CT

	**Disease-Free Survival**						**Overall Survival**					
	**PLN-/PALN-** (*n* = 71)		**PLN + /PALN-** (*n* = 45)		**PALN + ** (*n* = 32)		**PLN-/PALN-** (*n* = 71)		**PLN + /PALN-** (*n* = 45)		**PALN + ** (*n* = 32)	
	HR (95% CI)	*p*-value	HR (95% CI)	*p*-value	HR (95% CI)	*p*-value	HR (95% CI)	*p*-value	HR (95% CI)	*p*-value	HR (95% CI)	*p*-value
Tumor SUVmax	1.04 (0.99–1.09)	0.133	1.10 (1.03–1.19)	**0.009**	0.96 (0.86–1.07)	0.463	1.08 (1.01–1.16)	**0.018**	1.11 (1.02–1.21)	**0.011**	0.98 (0.87–1.10)	0.685
Tumor MTV (/10 cc)	1.10 (1.01–1.20)	**0.033**	1.19 (1.03–1.38)	**0.019**	1.02 (0.93–1.12)	0.674	1.12 (1.00–1.25)	0.057	1.22 (1.03–1.46)	**0.025**	1.06 (0.98–1.16)	0.161
High tumor MTV	5.14 (2.15–12.31)	** < 0.001**	3.17 (1.02–9.83)	**0.036**	1.15 (0.40–3.27)	0.793	6.10 (1.89–19.70)	** < 0.001**	3.46 (0.96–12.50)	**0.045**	2.13 (0.59–7.70)	0.236
Tumor size	1.28 (0.96–1.71)	0.087	1.57 (1.05–2.34)	**0.027**	1.04 (0.75–1.43)	0.819	1.42 (0.99–2.05)	0.058	1.43 (0.91–2.25)	0.118	1.23 (0.88–1.73)	0.225
Nb of PLN fixation	N/A	N/A	1.30 (1.10–1.53)	**0.002**	1.05 (0.86–1.29)	0.641	N/A	N/A	1.35 (1.11–1.64)	**0.003**	1.04 (0.85–1.27)	0.713
PLN SUVmean	N/A	N/A	1.32 (1.06–1.65)	**0.014**	1.01 (0.75–1.37)	0.939	N/A	N/A	1.42 (1.11–1.80)	**0.004**	1.14 (0.81–1.59)	0.459
PLN SUVmax	N/A	N/A	1.18 (1.06–1.31)	**0.002**	1.03 (0.90–1.17)	0.693	N/A	N/A	1.22 (1.08–1.37)	**0.001**	1.06 (0.91–1.23)	0.432

*PLN* pelvic lymph node, *PALN* para-aortic lymph node, *HR* hazard ratio, *CI* confidence interval, *N/A* not applicable

HR for time to distant metastasis in patients without lymph node metastasis on [^18^F]FDG-PET/CT was 4.73 (CI 95% [1.55–14.44] p = 0.003) for high MTV values of the cervical tumor. HR for time to distant metastasis in patients with PLN uptake and without PALN uptake were 1.35 (CI 95% [1.08–1.67] p = 0.007) for the number of PLN with uptake and 1.97 (CI 95% [0.49–7.97] p = 0.334) for high MTV values of the cervical tumor. Tumor SUVmax was significantly associated with time to loco-regional relapse in the overall cohort (HR 1.05 CI 95% [1.00–1.10] p = 0.049), but not in subgroup analysis. High MTV values of the cervical tumor also correlated with time to locoregional relapse in [^18^F]FDG-PET/CT node-negative patients (HR 5.18 [1.72–15.60] p = 0.001, but not in [^18^F]FDG-PET/CT node-positive patients. The number of PLN with uptake was associated with time to locoregional relapse in patients with PLN uptake but without PALN uptake (HR 1.31 CI 95% [1.08–1.59] p = 0.007). No significant association between tumor metabolic or lymph node metrics and time to distant or locoregional metastasis was found in patients with PALN uptake.

## Discussion

**Fig. 2:**
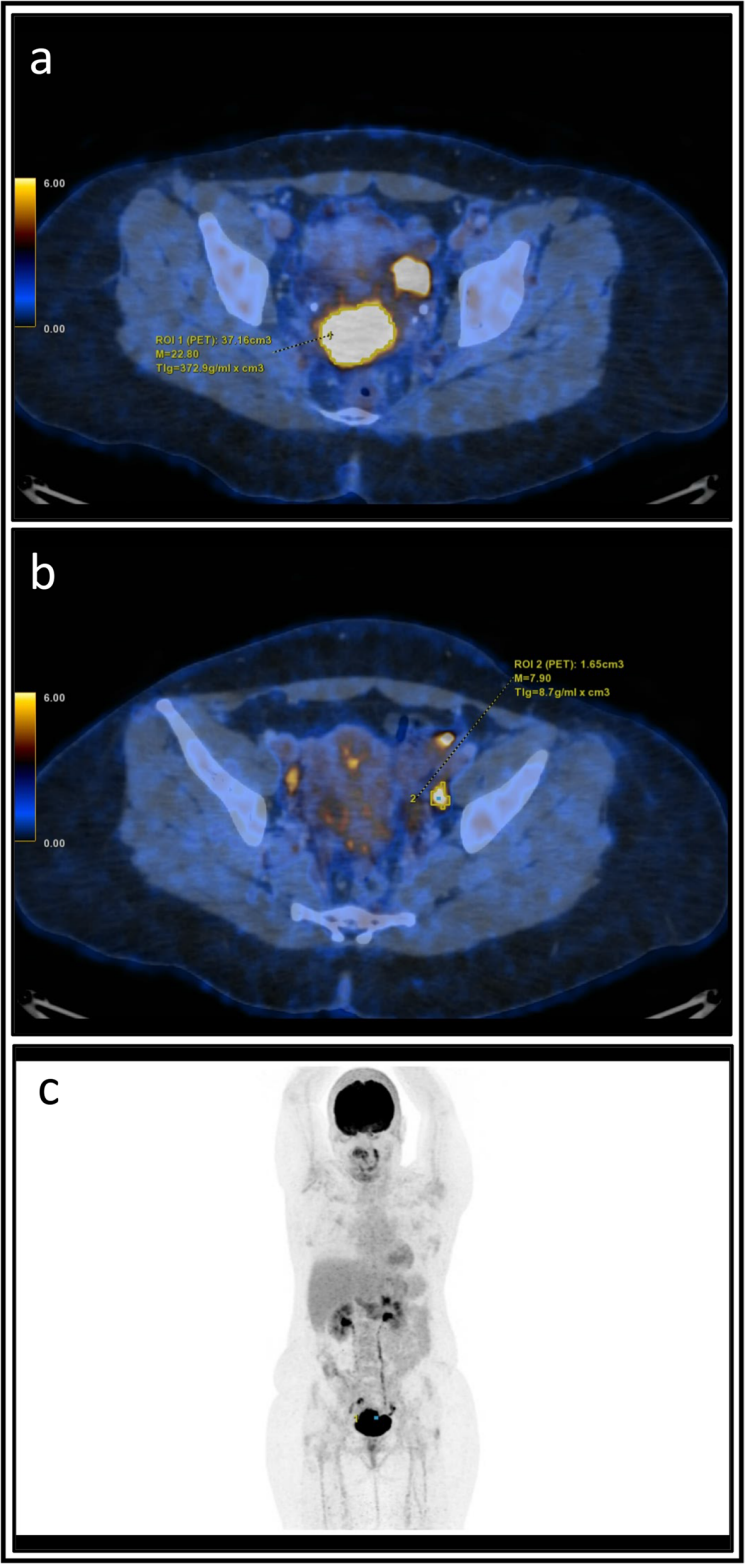
PET images of a patient presenting with a cervical tumor with left pelvic lymph node involvement. **a**) cervical tumor contouring with semi-automatic tumor thresholding method at 40% of the SUVmax (SUVmax 22.8; TLG 372.9 g/ml*cm^3^; MTV 37.16 cm^3^); **b**) the same procedure with a left pelvic involved lymph node with SUVmax 7.9; TLG 8.7 g/ml*cm^3^; MTV 1.65 cm^3^; **c**) Maximum intensity projection showing the absence of distant metastasis

PET/CT provides non-invasive information on what to expect from disease evolution according to the pattern of metabolic locoregional uptake (Fig. 2). We studied the extent to which tumor and lymph node metabolic activity determines recurrence risk. As demonstrated in previous series [10–13], higher tumor metabolic uptake was found in patients with lymph node involvement. The HR for disease recurrence, distant failure and death were significantly increased in patients with high MTV of the cervical tumor and in patients with a higher number of PLN fixations. Metabolic parameters did not predict prognostic outcome in patients with PALN metastasis. The prognostic value of PET/CT tumor and lymph node metabolic activity disappeared with PALN positivity, which was associated with distant failure in nearly half of patients.

High tumor metabolic activity has been correlated with poor prognostic factors such as high grade, poorly differentiated histology, depth of invasion, tumor size and lymph node involvement [6, 8, 10, 12, 16, 17]. In patients without lymph node spread, both SUVmax and MTV of the cervical tumor were correlated with shorter survival, but only high MTV values of the cervical tumor were associated with distant failure in our series. The prognostic value of tumor [^18^F]FDG uptake within tumor sites on pretreatment PET/CT has been shown in several reports [11, 18]. A recent meta-analysis demonstrated that patients with high tumor or lymph node SUVmax are at higher risk of adverse events or death [19]. In a previous study by our group, we showed that cervical metabolic activity was the main predictive factor of OS in patients with no para-aortic extension [6]. In the present series, our results also show an association between high SUVmax and locoregional recurrence risk in the overall cohort. Among metabolic parameters, high MTV of the primary tumor significantly increased the risk of locoregional, distant failure, and death. Volume-based metabolic parameters were associated with outcome after CRT in several studies [20–22]. Our results confirm those from a recent meta-analysis comprising 660 patients from 12 studies, which showed an increased risk of recurrence and death in patients with high values of MTV and TLG [22]. All except one of the studies included showed a survival effect of volumetric parameters in multivariate analysis, including additional prognostic, clinical and PET/CT parameters [22]. The authors suggested that cervical intratumor heterogeneity is associated with the underlying tumor biology, such as variable hypoxia, cellular proliferation, and blood flow, resulting in varying levels of [^18^F]FDG avidity. A correlation between volume-based parameters and both size and uptake distribution within the tumor was also found [23].

The method of measuring MTV varies across studies and a wide range of different MTV prognostic cut-off values have been reported [20]. Miller et al. reported shorter DFS and OS in node-negative patients with MTV ≥ 60 cm^3^ treated with CRT [20]. Chung et al. found that preoperative MTV ≥ 23.4 ml was an independent factor for DFS in multivariate analysis in patients treated with radical surgery [24]. Sun et al. also reported that MTV values above 53.75 ml were an independent factor for OS [25]. Other series failed to demonstrate a significant prognostic value of metabolic parameters when compared to other clinical prognostic factors such as tumor size or lymph node involvement in multivariate analysis [26]. Even if there are several prognostic MTV cut-off values and various methods for estimating and interpreting MTV, high MTV values have more impact on survival than SUVmax [27].

According to data provided by several series, high lymph node metabolic activity portends an increased risk of recurrence and death in patients with LACC treated with CRT [8, 28]. Kidd et al. showed in a prospective study that the risk of pelvic recurrence is significantly related to high PLN SUVmax but not to cervical tumor SUVmax, although their study also included higher levels of lymph node spread [8]. Nakamura et al. also found that high PLN SUVmax were associated with decreased DFS and OS in patients treated with CRT for cervical cancer with locoregional spread confined to the pelvis [29]. Another series provided evidence for the independent predictive value of the ratio [PLN SUVmax]/[cervical tumor SUVmax] for the risk of recurrence in the multivariate analysis of patients with pelvic and/or para-aortic extension [30]. Our results also showed a significant association between PLN SUV mean and survival in patients with no para-aortic extension on PET/CT. However, multivariate analysis showed that only MTV of the cervical tumor and the number of PLN with uptake remained as significant predictors of survival.

Our results are consistent with other studies that found MTV of the cervical tumor and PALN metastases to be the best predictors of OS [31]. The prognostic value of metabolic metrics disappeared in the presence of PALN involvement. Furthermore, PALN uptake avidity was not associated with prognostic outcome, as PALN involvement was itself more predictive of distant failure and death than any metabolic parameter of the tumor or lymph node. In contrast with our results, an association between OS and para-aortic SUV and TLG values was reported in a retrospective study including 68 patients with PALN extension [32]. Another study identified an SUVmax threshold above 3.3 of the PALN as a significant factor for OS [33]. PALN metastasis is a major outcome determinant, with decreased 5-year OS at approximately 30%, and a majority of patients with distant recurrent disease. Information on PALN status enables treatment intensification by extension of the radiotherapy field and the optional addition of adjuvant chemotherapy. In our series, we only found 3 patients (4.2%) with isolated PALN involvement, which is lower than other reports of patients with no PLN extension [12, 34, 35].

This and other studies indicate that MTV of the cervical tumor and the number of PLN fixations are independent factors for OS. However, to date, few studies have addressed the recurrence risk of functional PET/CT parameters according to the level of lymph node disease in LACC prior to CRT. High MTV values of the cervical tumor were associated with decreased DFS and OS in patients with disease confined to the pelvic region. They were also related to time to distant and locoregional recurrence in PET/CT lymph node-negative patients. Even if SUVmax has been widely and systematically used to define cervical tumor metabolic activity, MTV is more accurate to estimate relapse risk. Pretreatment PET/CT should include MTV measurement in routine practice in PALN-negative patients in order to tailor follow-up and intensify treatment modalities or favor inclusion in clinical trials for patients with high MTV values.

The main limitation of the study is its retrospective design, which could have induced a selection bias in the patient population. Additionally, dose levels and radiotherapy techniques have evolved during the last years and can influenced survival results. The long inclusion period can explain the lower survival rates than those reported in the EMBRACE studies [36]. This is the first study to estimate specifically cervical tumor and lymph node metabolic parameters to define the risk of distant failure depending on the pattern of retroperitoneal lymph node metastasis. A major strength of the study is lymph node surgical staging of all patients included in this series. To increase the reproducibility of metabolic parameter measurements, to facilitate their use in daily practice and to limit inter-observer variability, dedicated artificial intelligence thresholding software could prove valuable in the future.

In conclusion, MTV of the cervical tumor and number of PLN fixations were the best predictors of survival. MTV correlated with locoregional and distant failure in patients with LACC without lymph node involvement and the number of PLN fixations was associated with locoregional and distant failure in patients without PALN extension. The prognostic value of metabolic metrics disappeared with PALN positivity, which was associated with distant failure in nearly half of patients. Our results and previous studies demonstrate that MTV of the cervical tumor is a more accurate parameter than SUVmax to predict survival outcome and should be implemented in routine practice.

## Data Availability

The datasets generated during and/or analyzed during the current study are available from the corresponding author on reasonable request.

## Abbreviations

LACC: Locally Advanced Cervical Cancer
PET/CT: Positron Emission Tomography/computed Tomography
CT: Computed Tomography
MRI: Magnetic Resonance Imaging
CRT: Chemoradiotherapy
[^18^F]FDG-PET/CT: Fluorodeoxyglucose-PET/CT
PALN: Paraaortic lymph node
Gy: Gray
IMRT: Intensity-Modulated Radiation Therapy
PLN: Pelvic Lymph Node
SUVmax: Maximum Standardized Uptake Value
EANM: European Association of Nuclear Medicine
SUVmean: Mean Standardized Uptake Value
MTV: Metabolic Tumor Volume
TLG: Total Lesion Glycolysis
CI: Confidence Interval
DFS: Disease-Free Survival
OS: Overall Survival
HR: Hazard Ratio
